# Demographic, Clinical Profile of Rheumatoid Arthritis Patients and Their Association with Disease Severity in Ghana

**DOI:** 10.1155/2024/6639079

**Published:** 2024-01-12

**Authors:** Tonnies Abeku Buckman, Samuel Asamoah Sakyi, Kwame Yeboah-Mensah, Maxwell Hubert Antwi, Isaac Darban, Lawrence Owusu-Brenya, Joseph Yorke, Andy Opoku Boateng, Ebenezer Senu, Albert Dompreh, Akwasi Minnah Addei, Richard Boateng, Ortis Yankey, Samuel Tandoh

**Affiliations:** ^1^Department of Medical Laboratory Science, KAAF University College, Buduburam-Gomoa East District, Ghana; ^2^Department of Molecular Medicine, School of Medicine and Dentistry, Kwame Nkrumah University of Science and Technology, Kumasi, Ghana; ^3^Department of Medicine, Komfo Anokye Teaching Hospital, Kumasi, Ghana; ^4^Department of Medical Laboratory Sciences, Koforidua Technical University, Koforidua, Ghana; ^5^Department of Surgery, School of Medicine and Dentistry, Kwame Nkrumah University of Science and Technology, Kumasi, Ghana; ^6^Department of Microbiology, Komfo Anokye Teaching Hospital, Kumasi, Ghana; ^7^Department of Biological Sciences, Kwame Nkrumah University of Science and Technology, Kumasi, Ghana; ^8^School of Geography and Environmental Science, University of Southampton, Southampton, UK; ^9^University Clinic, University of Education, Winneba, Ghana

## Abstract

**Background:**

Rheumatoid arthritis (RA) is one of the frequent chronic, systemic, inflammatory autoimmune disorders with an estimated global prevalence of 1%. RA leads to joint destruction and disability if left untreated. Ghana has seen very few studies on RA, and little is known about the disease's severity and related variables. This study sought to characterize the clinical presentation and determine disease severity and associated risk factors with disease severity among RA patients in a tertiary hospital in Ghana.

**Methods:**

This cross-sectional study was conducted between September 2020 and August 2021. This study included 56 consecutively consenting RA patients from the Komfo Anokye Teaching Hospital orthopaedic unit. Diagnosis of RA was based on the updated American College of Rheumatology/European League Against Rheumatism (ACR/EULAR) 2022 rheumatoid arthritis classification criteria by a rheumatologist. A study questionnaire was used to gather participant demographics and clinical features, and results from the laboratory were taken from the patients' charts and medical records. The patients' disease severity was evaluated based on the rheumatoid arthritis disease activity score, which is based on a 28-joint count (DAS28), and their functioning was evaluated using the modified health assessment questionnaire.

**Results:**

The participants' mean age was 51.25 ± 13.22 years. Out of the total participants, 46 were females, and 10 were males (female-to-male ratio 4.6 : 1). Moreover, 37.50% had arthritis of the hand; 5.30% had severe disease, and 94.60% were not severe. A majority (76.80%) were on methotrexate medication. The most frequently involved joints were the knee (42.90%), wrist (32.10%), and elbow (12.50%). There was no statistically significant association with disease severity and a functional status score of >0.5 (cOR: 10.60, 95% CI (0.52-217.30); *p* = 0.124). In addition, marital status (*p* = 0.04), disease duration (*p* = 0.04), family complaints (*p* = 0.02), and ESR (*p* = 0.03) were significantly associated with disease severity.

**Conclusion:**

RA is predominant among elder populations and females. Disease duration, family complaints, and ESR are associated with disease severity. The findings of this study call for interventions towards ensuring early diagnosis of RA among high-risk populations to enhance good management practices.

## 1. Introduction

Rheumatoid arthritis is an autoimmune disease associated with chronic inflammation that progressively causes extensive damage to the joints of the body [1]. Studies have demonstrated that extra-articular presentations such as extensive bone and cartilage degradation and systemic manifestations seen among RA patients are associated with the severity of RA [2]. Anticyclic protein antibodies, rheumatoid nodules, and rheumatoid factor are among the other clinical manifestations that have been linked to the severity of RA in Caucasians and Asians [2, 3]. Poor prognosis factors for RA include persistently moderate or high disease activity (after csDMARD therapy) based on composite measures such as joint counts despite csDMARD therapy, high levels of acute phase reactants, high counts of swollen joints, presence of RF and/or ACPA, especially at high levels, early erosions, and failure of two or more csDMARDs. [4]. However, in Africa, delayed RA diagnosis is a key contributor to severe RA since most potential RA patients fail to report to hospitals for early diagnosis and treatment as many associate RA with superstitions [5, 6]. Although few studies have demonstrated low to mild RA in Africa, current studies have demonstrated the severity of RA in some parts of Africa [7]. Additionally, these reports of the severity of RA in some parts of Africa have been associated with some clinical presentations [6]. Studies have shown that most clinical presentations maybe influenced by genetic and lifestyle factors and, hence, may be population specific [8, 9]. Furthermore, investigating the clinical presentation of RA patients is necessary to initiate early and appropriate treatment guidelines and alleviate or prevent severe RA. Although the clinical presentation of RA has been associated with severe RA in other populations, no studies have investigated the clinical presentations of RA and their association with severe RA in Ghana. Thus, the purpose of this study is to examine the medical profile of RA and its relationship to RA severity in the Ghanaian population.

## 2. Method

### 2.1. Study Design and Setting

A cross-sectional study was conducted between September 2020 and August 2021.

Patient recruitment was done at the rheumatology units of Komfo Anokye Teaching Hospital (KATH) and Kumasi, Ghana. KATH, the second largest hospital in Ghana, is a 1200-bed facility in the Kumasi Metropolis. Kumasi is the second major city in Ghana and has a projected population of 4,780,380. The Rheumatology unit provides healthcare services to both in and outpatients with rheumatologic and autoimmune conditions.

### 2.2. Study Population

Patients who visited the clinic for rheumatology between September 2020 and August 2021 were qualified for participation. Patients included in this study (1) were diagnosed with RA by using the 2010 ACR/EULAR classification key [10], (2) were 18 years of age and above, and (3) gave their informed consent to be part of this study [11]. Any patient with undifferentiated arthritis or mixed connective tissue ailment was excluded.

### 2.3. Data Collection

Questionnaires were administered to obtain sociodemographic data from the participants.

Marital status, educational attainment, work position, age, sex, and history of alcohol and tobacco use are among the data gathered. Clinical information on the most common symptoms of RA, such as joint ache and swelling, stiffness in the morning, hyperthermia and weight depreciation, back pain, extra-articular manifestation, length of time since diagnosis, medication currently taken, family history, and existence of other chronic illnesses, were gathered and obtained from the hospital's archives. Weight was measured in the upright position to the nearest 0.1 kg using a calibrated balance beam scale. Height was measured (subjects stood erect, barefoot, with feet together, and looking forward) to the nearest 0.1 m using a measuring tape. Body mass index (BMI) was calculated using the equation BMI = weight(kg)/height^2^(m). A BMI < 18.5 kg/m^2^ was deemed underweight, 18.5–24.9 kg/m^2^ was regarded normal, and 25–29.9 kg/m^2^ was considered overweight [1]. Blood pressure was measured with an automated blood pressure apparatus (Omron MX3-Omron Matsusaka Co., Ltd., Japan) from the right arm after the subject had been made to sit for at least five minutes. The average of the two readings taken five minutes apart was recorded.

In this study, laboratory data for ESR levels, haemoglobin count, C-reactive protein (CRP), and rheumatoid factor (RF) were gathered. Positive or unfavourable qualitative reports were made on RF. The immunoassay analyzer (AQT90) was used to quantify CRP. The results were classified as elevated or normal, with <3 mg/L representing normal values for whole blood. If an individual's ESR was greater than 30 mm/h for females and more than 20 mm/h for males, it was deemed elevated for those over 50. The elevated ESR threshold for individuals aged 50 years or older was defined as >20 mm/h for females and >15 mm/h for males [12].

The World Health Organisation (WHO) criteria were followed in the classification of haemoglobin levels, which were classified as mild anaemia (11.0–13.0 g/dL for males and 11.0–12 g/dL for females), moderate anaemia (8.0–11.0 g/dL for both sexes), or severe anaemia (<8.0 g/dL for both sexes) [12]. Utilising the modified health assessment questionnaire (MHAQ), patients' performance disabilities were evaluated. The range of the MHAQ overall score is 0.0 to 3.0. Mild (MHAQ < 1.3), moderate (1.3 < MHAQ < 1.8), and severe (MHAQ > 1.8) are the three categories for the MHAQ scores. The average score of participants was calculated, and this allowed for the classification of each participant's score as either high or low. The MHAQ total score ranges from 0.0 to 3.0. The MHAQ scores are categorised as mild (MHAQ < 1.3), moderate (1.3 < MHAQ < 1.8), and severe (MHAQ > 1.8). The questionnaire asks about how easy their daily activities are, as well as a disability assessment exercise and questions about observed patient satisfaction with routine daily living activities [13]. Each participant's score was categorised as either high or low based on the frequencies of their scores, with an average score of 0.5 being utilised. In addition to questions about observed patient satisfaction with routine activities of daily life and a reported change in difficulty, the questionnaire asks participants how easily they completed everyday tasks, which is an assessment of their impairment. BRAF-NRS were used for fatigue assessment [13], with individuals who had a mean value of 5 or above were deemed to be fatigued. The rheumatoid arthritis disease activity score, which has been approved for classifying diseases, was used to assess the intensity of the RA. The formula for calculating the DAS28 is “DAS28 = [0.56×√(Tender joint count 28)] + [0.7 × In (Erythrocyte sedimentation rate)] + [0.28×√(Swollen joint count 28)] + [0.014 × (Global health)]”. DAS28 (<3.2) means low disease activity, DAS28 ≥ 3.2 but ≤5.1 indicates moderate disease activity while a high disease activity score is >5.1 indicating a severe ailment [14].

### 2.4. Data Analysis

The data were exported from Excel version 16 into SPSS version 16.0. Means and standard deviations were used to summarise continuous data with a normally distributed distribution, and medians and interquartile ranges (IQRs) were used for data that were not normally distributed. As a result of the collection of qualitative data, categorical statistics were summarised as frequencies and percentages. The sociodemographic variables, clinical features, and laboratory features were among the exploratory variables. Every factor from the bivariate analysis was taken into account in the multivariable analysis model. Though having a *p* value > 0.05, confounding variables like literacy rate and BMI were kept in the last model.

## 3. Results

In all, 56 participants diagnosed with RA were enrolled in the study. The participants' average age was 51.3 ± 13.2 years, where about 25.0% were older than 60 years, 30.4% were in the 51-60 years age group, 19.6% each were in the 41-50 years age group and 30-40 years age group, and 5.4% were within less than 30 years old. Females were 82.1%, and males made up the remaining 17.9%. Also, 64.3% were married, and 35.7% of them were single. About 58.9% were informally employed, while 16.1%, 14.3%, and 10.7% were formally employed, unemployed, and retired, respectively. The ethnicity of participants was as follows: Akan's formed 58.9% of the participants, while 16.1%, 14.3%, 7.1%, and 3.6% were Ewe, Ga, Northerner, and Krobo, respectively. Those who had not received formal education were 25.0%, while 51.8% had received primary education, 19.6% had received secondary education, and only 3.6% had received tertiary level of education. Christians were 96.4%, and Muslims formed the remaining 3.6%. Those who exercise frequently were 25.0%, while the 75.0% did not exercise or have irregular exercise. Moreover, only 1.8% were smokers, 5.4% had sickle cell disease, and 7.1% had history of cardiovascular disease (Table 1).

Nearly 19.6% experienced extra-articular manifestation (EAM), and 21.4% had deformed joints. Moreover, 12.5% had difficulty in breathing, and 19.6% had morning stiffness. Of the 56 participants, 26.8% had arthritis of more than 3 or more joints with 26.5% having general malaise as a symptom. As part of the symptoms recorded, 16.1%, 17.9%, 39.3%, 32.1%, and 32.1% had weight loss, fever of unknown origin, minimized feeling of back pain, neck pain, and severe back pains, respectively. Also, 23.2% had stiff spine, and 37.5% had arthritis of the hand (Table 2).

Higher portions of the participants, 76.8% and 57.1%, were taking methotrexate and folic acid, respectively, and few of the participants, 19.6%, 14.3%, 12.5%, and 39.3%, were on nifedipine, amlodipine, losartan, and prednisolone drugs, respectively (Table 3).

Many patients involved in the study were obese (46.4%) with few being underweight (3.6%). Moreover, 39.3% and 19.6% had normal systolic and diastolic pressure, respectively; 32.1% and 39.3% were in the prehypertensive stage for systolic and diastolic pressure, respectively. Also, 28.6% and 39.3% were in stage 1 hypertensive group for systolic and diastolic pressure, respectively. There was no participant for stage 2 hypertension for systolic pressure, but 1.8% were in stage 2 hypertension category for diastolic blood pressure. ESR level was elevated in 69.6% of the participants with the median ESR of 36.0 mm/hour. Fairly, many of the participants (55.4%) had mild anaemia, and 64.3% were positive for RF. From the total participants, 39 had their C-reactive protein measured, and 30.4% had elevated levels. The mean waist circumference was 35.79 ± 4.99 m, and the mean hip circumference measured was 40.09 ± 5.60 m (Table 4).

Table 5 summarises the disease duration and the functional disability of the participants. Many participants (85.7%) had the disease for more than 15 months, and 3.6% had experienced previous diagnosis of the condition. With MHAQ levels, 58.9% were having normal, and 7.1% were having severe levels. In addition, 8.9% had family complaints (Table 5).

**Figure 1:**
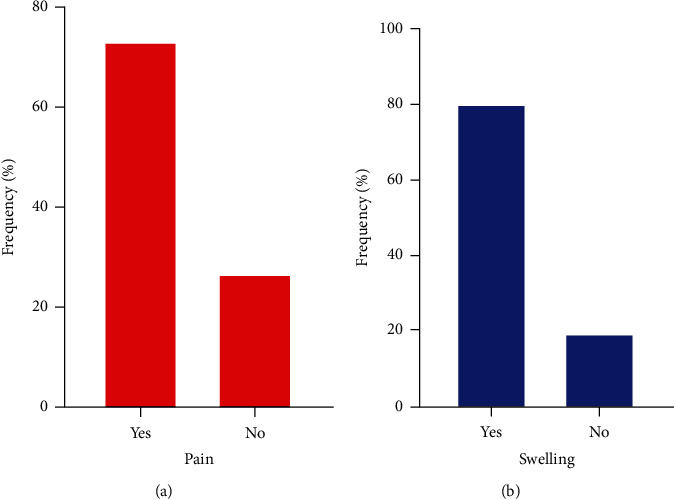
Symptoms of pain and the signs of swelling. (a) Proportion of participants who experienced or did not experience pain at the joints. (b) Proportion of participants who had or did not have a swollen joint.

A greater number (74%) of study participants showed symptoms of pain, and a greater proportion (80%) had swollen joints (Figure 1).

**Figure 2:**
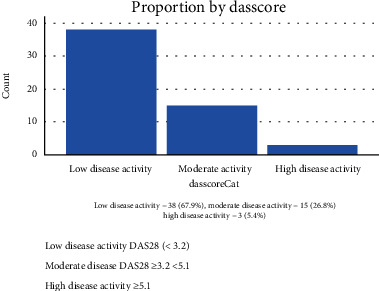
Disease activity score based on 28 joint counts and ESR (DAS28-ESR) among study participants.

A greater number (67.9%) of study participants had low disease activity, 26.9% had moderate disease activity, and 5.4% showed high disease activity using DAS28 (Figure 2).

**Figure 3:**
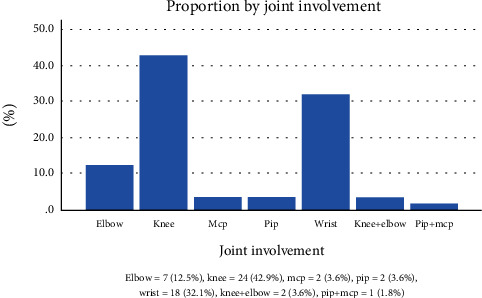
Rheumatoid arthritis by joint involvement.

The knee (42.9%), wrist (32.1%), and elbow (12.5%) were the most involved joint in the study population (Figure 3).

The joints swollen and with pain were accessed from among the participants. 41 were having pains at some joints, and 45 experienced swelling at the joints. Among 41 participants who were in pain, the majority (39.0%) complained of knee aches, a few (2.4%) had pains at the metacarpophalangeal (MCP) joint, and the same proportion had pain at both their MCP and proximal interphalangeal (PIP) joint. From a total of 45 participants who experienced swelling at their joints, 37.8% had swollen wrists, a few (2.2%) had PIP involved, and the same proportion had PIP and MCP swollen (Table 6).

A significant correlation was found between disease severity and marital status (*p* = 0.041), disease duration (*p* = 0.040), and family history of RA (*p* = 0.019**)**. No significant correlation was found between the remaining variables and the intensity of the condition (Table 7).

Characteristics such as hip circumference, waist circumference, age of participants, BMI, Hb, and CRP were not significantly correlated with disease severity (*p* > 0.05). A significant association was found between disease severity and ESR (*p* = 0.026) and family complaints (*p* = 0.010). For every mm/hour increase in the ESR of participants, there is a significant 7.7% increase in disease severity. Also, as compared to patients with no family complaints, patients with family complaints have 33 times the chance of having increased disease severity (Table 8).

## 4. Discussion

Rheumatoid arthritis is still a global health concern as it causes joint damage as well as long-term disability. The goal of the study was to assess the clinical presentation, measure the severity of the disease, and identify the risk factors for severe rheumatoid arthritis (RA) in patients attending a tertiary hospital in Ghana. Clinical, lifestyle, and sociodemographic characteristics were assessed. The study did not see any major significant observations of these characteristics on the disease condition except that rheumatoid arthritis was less recorded among the younger age group as compared to the older age group in this study with a mean age of 51.25. This study is in support of studies that have linked rheumatoid arthritis to age [15, 16].

The clinical diagnostic symptoms that define RA in the study were extra-articular manifestation, deformed joint, difficulty in breathing, stiffness in the morning, arthritis of 3 or more joints, and general malaise which included fever, back pains, sniff spine, and neck pain. Among these symptoms, arthritis of the hand was seen to be the prevailing symptom among the participants even though it was insignificant in correlating to severity. Ochola et al. [6], in their studies, reported that the hand joints were frequently affected by the illness, which is consistent with our findings that hand arthritis was a prevalent symptom. A very low percentage of research participants—nearly 5%—were classified as having an intense disease, which suggests either early disease identification or a good prognosis (disease remission). Significant characteristics that saw a correlation with disease intensity were marital status (*p* = 0.041), how long a participant has had the disease (*p* = 0.04), family complaints (*p* = 0.019), and DAS28 score (*p* ≤ 0.00001). Unfortunately, the remaining laboratory and clinical features that were assessed in the study were not significantly correlated with disease intensity. Marital status has generally been associated with RA health status and again linked with the level of adjustment in the marriage being more important to consider than simply whether or not one is married. Analytical findings from a study that compared three groups assessed on marital status and level of marital adjustment reported that nondistressed married participants tended to have better health than both distressed married and unmarried participants. This study was limited to only marital status and did not go further to know the degree of challenges in the marriage. It is presumed that family pressure with persistent complaints worsens disease severity as compared to nondistressed marriage participants who get the needed support to be both psychologically and mentally sound which mostly promotes disease remission [17, 18]. Also, as compared to patients with no family complaints, patients with family complaints have 33 times the chance of having increased disease severity.

Even though the DAS28 score was recorded mostly among the nonsevere RA patients, it was seen to be significant in the intensity of the disease condition as a high DAS28 score was equally assessed on some severe RA patients. A study done by Ochola et al. [6] reported that a substantial correlation was found between the DAS28 score and the intensity of the RA in the current study, as well as in theirs, with over half of the patients involved in the study having severe RA when the DAS28-ESR score was used. Singwe-Ngandeu et al. [19] have also reported a high DAS28-ESR score > 5.1 among most of their studied participants which again saw no major difference with the present study in significance to the disease severity. Findings by Lee et al. [15] found that while individuals in DAS28 remission may still have increased pain and disease progression in cases of sleep disturbances, exhaustion, and incapacity, heightened pain severity was not associated with joint deterioration. Again, only 1 (1.9%) of the patients involved was a smoker, and the association between smoking and RA was not significant. This finding contradicts the studies of Seror et al. [20] where active smoking was significantly correlated with an elevated risk of RA. In the studies conducted by Chang et al. [8] and also by Ishikawa and Terao [21], smoking was found to be linked with an elevated risk of RA, and smoking can also affect the drugs in RA treatment. The difference between our findings and theirs could be linked to the fewer number of our participants who were smokers.

RA disease severity was an independent risk factor for hypertension, anaemia, body mass index, inflammation, and cardiovascular disease in the study since there were no significant associations between RA severity and these clinical characteristics. Masuda et al. [22] reported that the disease duration of RA is a separate risk factor for cardiovascular diseases which was not too different from what the present study observed. Significant unidirectional alterations in receptor expression in RA did not correlate with longer disease duration as reported by Alshevskaya et al. [23] which contradicts our finding where severity was linked to the time frame of the disease condition. Although the trend was very weak [24], findings also indicated that disease intensity had a very slow downward trend over three years in their studies, and they ascribed the duration of care and participants being men as significant related factors with decreased severity of the ailment.

Assessments were conducted on the clinical features of RA based on joint involvement. Although the tiny joints of the wrist and knee were not statistically significant in terms of the disease's intensity, most patients involved in this study reported joint discomfort and swelling. Zhao et al. [25], in their studies, also reported wrist and ankle joint involvement in RA which was not too different from this current study. Another study by Kanazawa et al. [26] also corroborated our findings in their reports with the wrist joint being again the most affected joint involvement in RA patients. Wrist joint involvement has also been reported by Skakic V. and Skakic C. [27] to be the main predominant joint involvement in RA patients which again was in accordance with our study findings.

Features like participants' age, circumferences of the waist and hip, and haemoglobin levels were not linked to the intensity of the condition. C-reactive protein as a clinical inflammatory marker in this study for the disease progression was not also seen as a significant characteristic which was supported by similar findings from Fleischmann et al. [28]. The association between disease severity and ESR (*p* = 0.026) was statistically significant. For every mm/hour increase in the ESR of participants, a significant 7.7% increase in disease severity exists according to the present study findings. This finding is in support of a study that has correlated the disease severity to ESR increase [29]. ESR has also been reported to increase RA severity and progression [30].

The limitations of this study are the small sample sizes for part of the study variables; higher sample sizes may be required in future research to accentuate these findings. Additionally, only at the initial contact was the disease intensity assessed necessitating further prospective research to identify additional relevant factors.

## 5. Conclusion

RA is predominant among elderly populations and females. Knee and wrist joint involvement are the predominant joint involvement in the RA subjects. Disease duration, family complaints, and ESR are linked with disease severity. This study's findings call for interventions towards prevention or early diagnosis of RA among high-risk populations to enhance good management.

## Figures and Tables

**Table 1:** Sociodemographic features of study participants.

Characteristic	Frequency (*n* = 56)	Percentage (%)
Ages		
<30	3	5.4
30-40	11	19.6
41-50	11	19.6
51-60	17	30.4
>60	14	25.0
Gender		
Males	10	17.9
Females	46	82.1
Marital status		
Single	20	35.7
Married	36	64.3
Ethnicity		
Akan	33	58.9
Ewe	9	16.1
Ga	8	14.3
Krobo	2	3.6
Northern	4	7.1
Employment status		
Unemployed	8	14.3
Formal	9	16.1
Informal	33	58.9
Retired	6	10.7
Education		
Noneducation	14	25.0
Primary	29	51.8
Secondary	11	19.6
Tertiary	2	3.6
Religion		
Christian	54	96.4
Muslim	2	3.6
Exercise		
Yes	14	25.0
No	42	75.0
Smoking		
Yes	1	1.8
No	55	98.2
Sickle cell status		
Yes	3	5.4
No	53	94.6
Cardiovascular disease		
Yes	4	7.1
No	52	92.9
Variable	Mean ± S.D	
Age	51.25 ± 13.227	

**Table 2:** Response to clinical signs and symptoms of participants.

Symptoms	Frequency (*n* = 56)	Percentage (%)
Extra articular manifestation		
Yes	11	19.6
No	45	86.4
Deformed joint		
Yes	12	21.4
No	44	78.6
Deep breath		
Yes	7	12.5
No	49	87.5
Morning stiffness		
Yes	11	19.6
No	45	80.4
Arthritis of 3 or more joints		
Yes	15	26.8
No	41	73.2
Generalized malaise		
Yes	15	26.8
No	41	73.2
Arthritis of hand		
Yes	21	37.5
No	35	62.5
Weight loss		
Yes	9	16.1
No	47	83.9
Fever of unknown origin		
Yes	10	17.9
No	46	82.1
Low back pain		
Yes	22	39.3
No	34	60.7
Neck pain		
Yes	18	32.1
No	38	67.9
Back pain		
Yes	18	32.1
No	38	67.9

Abbreviation: EAM: extra-articular manifestation.

**Table 3:** Response to drug usage by participants.

Drug	Frequency (*n* = 56)	Percentage (%)
Methotrexates		
Yes	43	76.8
No	13	23.2
Nifedipine		
Yes	11	19.6
No	45	80.4
Amlodipine		
Yes	8	14.3
No	48	85.7
Folic acid		
Yes	32	57.1
No	24	42.9
Losartan		
Yes	7	12.5
No	49	87.5
Prednisolone		
Yes	22	39.3
No	34	60.7

**Table 4:** Clinical laboratory characteristics of study participants.

Characteristic	Frequency (*n* = 56)	Percentage (%)
Body mass index (kg/m^2^)		
Underweight	2	3.6
Healthy	11	19.6
Overweight	17	30.4
Obese	26	46.4
Systolic (mmHg)		
Normothermic	22	39.3
Prehypertensive	18	32.1
Stage 1 hypertension	16	28.6
Stage 2 hypertension	0	0.0
Diastolic (mmHg)		
Normothermic	11	19.6
Prehypertensive	22	39.3
Stage 1 hypertension	22	39.3
Stage 2 hypertension	1	1.8
Erythrocyte sedimentation rate (mm/h)		
Normal	17	30.4
Elevated	39	69.6
C reactive protein (mg/dL)	**(n =39)**	
Normal (≤3)	22	56.4
Elevated (>3)	17	43.59
Haemoglobin level(g/dL)		
Anaemic	0	0.0
Mild anaemia	31	55.4
Normal	25	44.6
Rheumatoid factor (IU/mL)		
Negative	20	35.7
Positive	36	64.3
Variable	Mean's/median (IQR)	
Waist circumference (cm)	35.79 ± 4.99	
Hip circumference (cm)	40.09 ± 5.60	
ESR	36.0(29.25-51.25)	

Abbreviation: ESR: erythrocyte sedimentation rate.

**Table 5:** Disease duration and functional disability of study participants.

Variable	Frequency (*n* = 56)	Percentage (%)
Disease duration		
<6 months	2	3.6
6-10 months	2	3.6
11-15 months	4	7.1
>15 months	48	85.7
MHAQ		
Normal	33	58.9
Moderate	19	33.9
Severe	4	7.1
Family complaints		
Yes	5	8.9
No	51	91.1

**Table 6:** Clinical features of rheumatoid arthritis by joint involvement.

Joint involved	Pain (*n* = 41)	Swelling (*n* = 45)
	Frequency (%)	Frequency (%)
Elbow	6 (14.6)	7 (15.6)
Knee	16 (39.0)	15 (33.3)
MCP	1 (2.4)	2 (4.4)
PIP	2 (4.9)	1 (2.2)
Wrist	13 (31.7)	17 (37.8)
Knee+elbow	2 (4.9)	2 (4.4)
PIP+MCP	1 (2.4)	1 (2.2)

Abbreviations: PIP: proximal interphalangeal joint; MCP: metacarpophalangeal joint.

**Table 7:** Association of the characteristics and disease severity.

Characteristic	Not severe (53), *n* (%)	Severe (3), *n* (%)	*p* value
Ages (years)			0.174
<30	2 (3.8)	1 (33.3)	
30-40	10 (18.9)	1 (33.3)	
41-50	11 (20.8)	0 (0.0)	
51-60	16 (30.2)	1 (33.3)	
>60	14 (26.4)	0 (0.0)	
Gender			0.452
Males	9 (17.0)	1 (33.3)	
Females	44 (83.0)	2 (66.7)	
Marital status			0.041
Single	17 (32.1)	3 (100)	
Married	36 (67.9)	0 (0.0)	
Employment status			0.530
Unemployed	8 (15.1)	0 (0.0)	
Formal	9 (17.0)	0 (0.0)	
Informal	30 (56.6)	3 (100)	
Retired	6 (11.3)	0 (0.0)	
Education			0.719
Noneducated	2 (3.8)	0 (0.0)	
Primary	14 (26.4)	0 (0.0)	
Secondary	27 (50.9)	2 (66.7)	
Tertiary	10 (18.9)	1 (33.3)	
Smoking			0.946
Yes	1 (1.9)	0 (0.0)	
No	52 (98.1)	3 (100)	
Cardiovascular disease			0.797
Yes	4 (7.5)	0 (0.0)	
No	49 (92.5)	3 (100)	
B.M.I (kg/m^2^)			0.526
Underweight	2 (3.8)	0 (0.0)	
Healthy	11 (20.8)	0 (0.0)	
Overweight	15 (28.3)	2 (66.7)	
Obese	25 (47.2)	1 (33.3)	
ESR (mm/h)			0.330
Normal	17 (32.1)	0 (0.0)	
Elevated	36 (67.9)	3 (100)	
HB (g/dL)			0.582
Anaemic	0 (0.0)	0 (0.0)	
Mild anaemia	29 (54.7)	2 (66.7)	
Normal	24 (45.3)	1 (33.3)	
RF			0.288
Negative	18 (34.0)	2 (66.7)	
Positive	35 (66.0)	1 (33.3)	
Disease duration (months)			0.040
<6 months	2 (3.8)	0 (0.0)	
6-10 months	1 (1.9)	1 (33.3)	
11-15 months	4 (7.5)	0 (0.0)	
>15 months	46 (86.8)	2 (66.7)	
MHAQ			
Normal	32 (60.4)	1 (33.3)	
Moderate	18 (34.0)	1 (33.3)	
Severe	3 (5.7)	1 (33.3)	
Family history of RA			0.019
Yes	3 (5.7)	2 (66.7)	
No	50 (94.3)	1 (33.3)	
Pain			0.615
Yes	39 (73.6)	2 (66.7)	
No	14 (26.4)	1 (33.3)	
Swelling			0.095
Yes	44 (83.0)	1 (33.3)	
No	9 (17.0)	2 (66.7)	

MHAQ: modified health assessment questionnaire.

**Table 8:** Clinical and laboratory factors associated with disease severity.

Variable	O.R (95% CI)	*p* value
Family history of RA		
No	1	
Yes	33.3 (2.312-480.4)	0.010
Hip circumference (cm)	0.88 (0.711-1.0)	0.239
Waist circumference (cm)	0.84 (0.642-1.1)	0.223
Age (years)	0.91 (0.822-1.0)	0.129
ESR (mm/h)	1.07 (1.009-1.2)	0.026
B.M.I (kg/m^2^)	0.97 (0.833-1.1)	0.761
HB (g/dL)	1.13 (0.595-2.1)	0.708
CRP (mg/dL)	21.29 (0.778-582.7)	0.070
MHAQ		
Mild	1	
Moderate	1.78 (0.105-30.12)	0.690
Severe	10.6 (0.52-217.3)	0.124

## Data Availability

The raw data used to support the findings of this study are available from the corresponding author upon request.
